# Functional annotation and importance of marine bacterial transporters of plankton exometabolites

**DOI:** 10.1038/s43705-023-00244-6

**Published:** 2023-04-25

**Authors:** William F. Schroer, Hannah E. Kepner, Mario Uchimiya, Catalina Mejia, Lidimarie Trujillo Rodriguez, Christopher R. Reisch, Mary Ann Moran

**Affiliations:** 1 Department of Marine Sciences, University of Georgia, Athens, GA 30602 USA; 2 College of Fisheries and Ocean Sciences, University of Alaska Fairbanks, Fairbanks, AK 99775 USA; 3 Complex Carbohydrate Research Center, University of Georgia, Athens, GA 30602 USA; 4 Department of Microbiology and Cell Science, University of Florida, Gainesville, FL 32611 USA

**Keywords:** Biogeochemistry, Microbial ecology

## Abstract

Metabolite exchange within marine microbial communities transfers carbon and other major elements through global cycles and forms the basis of microbial interactions. Yet lack of gene annotations and concern about the quality of existing ones remain major impediments to revealing currencies of carbon flux. We employed an arrayed mutant library of the marine bacterium *Ruegeria pomeroyi* DSS-3 to experimentally annotate substrates of organic compound transporter systems, using mutant growth and compound drawdown analyses to link transporters to their cognate substrates. Mutant experiments verified substrates for thirteen *R. pomeroyi* transporters. Four were previously hypothesized based on gene expression data (taurine, glucose/xylose, isethionate, and cadaverine/putrescine/spermidine); five were previously hypothesized based on homology to experimentally annotated transporters in other bacteria (citrate, glycerol, *N*-acetylglucosamine, fumarate/malate/succinate, and dimethylsulfoniopropionate); and four had no previous annotations (thymidine, carnitine, cysteate, and 3-hydroxybutyrate). These bring the total number of experimentally-verified organic carbon influx transporters to 18 of 126 in the *R. pomeroyi* genome. In a longitudinal study of a coastal phytoplankton bloom, expression patterns of the experimentally annotated transporters linked them to different stages of the bloom, and also led to the hypothesis that citrate and 3-hydroxybutyrate were among the most highly available bacterial substrates. Improved functional annotation of the gatekeepers of organic carbon uptake is critical for deciphering carbon flux and fate in microbial ecosystems.

## Introduction

The ocean microbiome plays a central role in mediating carbon and element cycles through its unique ability to process organic carbon dissolved in seawater [1–3]. Ultimately, bacteria take up and assimilate as much as half of marine net primary production (NPP) in the form of exometabolites derived from excretion and death of phytoplankton and other microbes [3, 4]. Given that current and future controls over this globally significant carbon flux are poorly understood, identification of the metabolites produced and consumed by ocean microbes is vitally important [5].

One approach to unraveling marine metabolite flux is through the application of transcriptomic and proteomic tools by which dynamics of the chemical environment can be gleaned from changes in the expression of microbial genes. Such approaches are easy to scale with advancements in sequencing and data sharing [6, 7] and have successfully addressed metabolite dynamics in various microbial systems such as model communities [8], phytoplankton blooms [9, 10], oligotrophic ocean regions [11, 12], and global-scale ocean surveys [13–15]. Transporter genes in particular are of value in such approaches because they are a cell’s interface with its environment and their expression can reveal the identity of available metabolites [16]. A key limitation to their use, however, is a dependence on accurate gene annotation to identify protein function. For most microbial transporters, the cognate substrate is still unknown. Others are annotated computationally based on homology [17–19], yet this is error prone when relationships to experimentally annotated genes are distant [20]. Indeed, transporters have a lower rate of successful annotation based on homology than catabolic enzymes [19].

Experimental confirmation of gene function is the gold standard for annotation, but is both time and resource intensive. Moreover, it is largely limited to cultured species for which genetic systems are available, leaving out much of the diversity represented in environmental bacteria. An alternate approach uses pooled transposon mutants whose fitness under defined selection pressure provides hypotheses of gene function [21–24]. This method requires only a minimal genetic system to introduce small DNA fragments (transposons) and a protein that catalyzes insertion of the transposons (transposase) into bacterial cells. A recent high-throughput advancement of this method, termed RB-TnSeq [25, 26], uses unique barcodes that link each transposon insertion site to the specific gene it disrupts, thereby allowing mutant pools to be analyzed for fitness through cost-effective amplicon sequencing. A wide taxonomic range of bacteria have been found amenable to RB-TnSeq library construction, resulting in hypothesis generation for gene functions that include stress response, metabolism, phage resistance, and transport [25, 27–29]. Hypotheses can be confirmed experimentally if targeted single-gene mutants are subsequently constructed, as for those predicting membrane proteins [28] and catabolic enzymes [30].

For a small number of well-studied model bacterial species, genome-wide mutant arrays have been constructed through painstaking targeted gene deletions to produce libraries of single-gene knockouts across the genome. Excellent tools for gene annotation, these arrayed libraries are currently available for species such as *Escherichia coli* [31], *Acinetobacter baylyi* [32], *Bacillus subtilis* [33, 34], and *Salmonella enterica* [35]. Pooled transposon mutant libraries have been used successfully as the starting material for such arrayed libraries [24, 36, 37], but require individual sequencing of tens of thousands of colonies to determine transposon insertion location.

Recently, a modification of the RB-TnSeq approach was used to create an inexpensive arrayed mutant library of the model marine bacterium *Ruegeria pomeroyi* DSS-3 [38]. The method took advantage of the ease of insertion site identification in RB-TnSeq libraries to map 270,000 barcodes to transposon insertions which were condensed into a library of 4991 mutants, similar to the approach used recently for the anaerobic gut microbe *Bacteroides thetaiotaomicron* [39]. *R. pomeroyi* is known for its ecological association with marine phytoplankton and ability to grow on plankton-derived metabolites [16, 40, 41], but to this point substrates of only four of the 126 putative organic compound influx transporters have been experimentally verified via knockout mutants: choline [42], dihydroxypropanesulfonate (DHPS) [41, 43], ectoine [44], and trimethylamine N-oxide [13]. Here we leveraged a set of 156 influx transporter mutants from the *R. pomeroyi* arrayed RB-TnSeq (arrayed-RB-TnSeq) library in high-throughput screens against 70 possible substrates to increase knowledge of transporter function. Resulting gene annotations were then applied to a longitudinal study of *R. pomeroyi* transcriptomes following introduction of the bacterium into Monterey Bay, CA, USA seawater on 14 dates during a dinoflagellate bloom [9] in the manner of an ecological invasion study [45]. The 13 newly verified transporter annotations provided insights into the metabolites serving roles as substrates to heterotrophic bacteria during a coastal bloom.

## Methods

### RB-TnSeq library generation and mapping

Full methods for generating and arraying the *R. pomeroyi* barcoded mutant library are provided in Mejia et al. [38]. Briefly, a pool of randomly barcoded transposon mutants was constructed according to Wetmore et al. [26] by conjugating *R. pomeroyi* DSS-3 with *E.coli* WM3064 containing the pKMW7 Tn5 barcoded library (strain APA766). The transposon insertion sites were subsequently linked to the unique barcodes through random barcode transposon-site sequencing (RB-TnSeq) [26]. RB-TnSeq was achieved by sequencing on an Illumina NovaSeq6000 with 150-bp paired-end reads (Novogene Co., Sacramento, CA). Bioinformatic analysis of the reads used custom scripts described in Wetmore et al. [26]. To construct the arrayed libraries, individual mutants were isolated on ½ YTSS solid medium amended with 100 µg ml^−1^ kanamycin. Colonies were picked after 2 d (Qpix2 automated colony picker; Molecular Devices, San Jose, CA) and arrayed into 384 well plates containing 80 µl of ½ YTSS + kanamycin broth. Plates were incubated at 30 ^o^C for 2–5 d until visible growth appeared and then replicated. Glycerol was added to a final concentration of 20% and plates were frozen at −80 ^o^C. To identify the mutant locations, barcodes were amplified in each well using a unique combination of indexed 16 forward and 24 reverse location primers, allowing indexing of the well position. These barcode amplicons were re-amplified with dual indexed Illumina adapters, and amplicons from this second round of PCR were pooled within a plate for sequencing [38]. Of the 270,510 unique barcodes originally present in the pooled RB-TnSeq library, the condensed RB-TnSeq arrayed library contained 4991 mutants covering 3048 of 4284 protein encoding genes with either 1 or 2 mutants, accounting for 71% of *R. pomeroyi*’s genes. From these, the first 156 putative organic compound influx transporter mutants identified in the arraying procedure were re-arrayed into two plates for subsequent screening (Table S1).

### Growth screens

Mutant cultures were pre-grown overnight in ½ YTSS broth with 50 μg ml^−1^ kanamycin. Screens were performed in L1 minimal medium [46] (10.17504/protocols.io.jvccn2w) modified to a salinity of 20 ppt and amended with ammonium (3 mM), kanamycin (50 μg ml^−1^), and phosphorus as PO_4_^3-^ at 36 μM. For the initial screen, overnight cultures of individual mutants (2 μl) were inoculated into 198 μl of modified L1 with a single substrate as the sole carbon source at 8 mM carbon. Plates were incubated at 25 ^o^C with shaking, and optical density (OD_600_) was read at intervals of 6–12 h using a SpectraMax M3 (Molecular Devices, San Jose, CA) until cultures entered stationary phase at ~24–48 h. Mutants exhibiting phenotypes in the initial screen were moved to the targeted screen in which 4 replicate 200 µl mutant cultures were prepared by inoculating 2 μl of washed (3x) overnight culture into 96 well plates containing 198 μl modified L1 medium and a single substrate at 8 mM carbon. As a positive control, four wells with the same medium were inoculated with washed overnight cultures of the pooled-RB-TnSeq library, used as a proxy for wild-type *R. pomeroyi* growth but harboring a transposon/kanamycin resistance gene insertion. Cultures were grown at 25 ^o^C in a Synergy H1 plate reader (BioTek, Winooski, VT, USA) shaking at 425 rpm for 68–72 h. OD_600_ readings were collected once each hour and corrected to a pathlength of 1 cm assuming a volume of 200 µl.

Mutant defect was identified by comparison to the OD_600_ achieved by the pooled-RB-TnSeq library (*n* = 4; ANOVA and TukeyHSD; *p* ≤ 0.05) (Table 1). Mutants with significantly lower OD_600_ on multiple substrates were regrown on rich medium to check for viability, and removed from further consideration if they broadly demonstrated poor growth; one mutant was removed after this viability check (SPO2952).

**Table 1:** Transporter identification based on growth and metabolite drawdown screens.

Transporter	Mutant	Substrate	Prediction	ΔOD (%)	95% CI (%±)	*p* adj	ΔDrawdown (%)	95% CI (%±)	*p* adj
*tctABC*	**Δ**SPO0184	Citrate	homology	99.1	12.4	1.1 × 10^–6^	99.3	3.6	5.4 × 10^–12^
*nupABC*	**Δ**SPO0378	Thymidine	novel	81.1	23.5	1.3 × 10^–5^	63.6	32.5	2.3 × 10^–3^
	**Δ**SPO0379	Thymidine	novel	81.5	23.5	1.3 × 10^–5^	83.0	32.5	5.6 × 10^–4^
*hpsKLM*	**Δ**SPO0591	DHPS	control	78.6	30.7	7.7 × 10^–4^	64.8	19.0	1.1 × 10^–4^
*glpVSTPQ*	**Δ**SPO0608	Glycerol	homology	79.6	13.0	5.3 × 10^–6^	78.8	2.8	2.1 × 10^–12^
*tauABC*	**Δ**SPO0674	Taurine	expression	91.8	20.6	1.5 × 10^–6^	–3.1	34.2	N.S.
	**Δ**SPO0676	Taurine	expression	92.8	20.6	1.4 × 10^–6^	49.1	34.2	1.1 × 10^–2^
*xylFGH*	**Δ**SPO0863	Glucose	expression	92.1	9.7	4.9 × 10^–7^	85.6	30.9	1.5 × 10^–3^
	**Δ**SPO0863	Xylose	expression	96.6	5.6	1.1 × 10^–8^	52.8	45.5	3.2 × 10^–2^
*betT*	**Δ**SPO1087	Choline	control	100.5	8.9	2.3 × 10^–7^	94.1	10.8	2.2 × 10^–5^
*uehABC*	**Δ**SPO1147	Ectoine	control	86.7	6.1	5.5 × 10^–8^	62.1	3.8	9.2 × 10^–3^
*nagTUVW*	**Δ**SPO1839	GlcNAc	homology	78.4	7.1	2.6 × 10^–7^	92.7	5.0	5.7 × 10^–8^
*iseKLM*	**Δ**SPO2357	Isethionate	expression	101.1	10.2	1.8 × 10^–9^	96.0	41.9	1.0 × 10^–3^
	**Δ**SPO2358	Isethionate	expression	104.2	10.2	1.6 × 10^–9^	69.8	41.9	5.3 × 10^–3^
*hbtABC*	**Δ**SPO2573	3-OH butyrate	novel	92.7	3.9	5.4 × 10^–10^	79.8	11.7	5.3 × 10^–5^
*dctMPQ*	**Δ**SPO2626	Fumarate	homology	99.1	3.3	2.7 × 10^–14^	98.0	7.9	1.6 × 10^–9^
	**Δ**SPO2626	Malate	homology	48.6	25.3	4.9 × 10^–4^	58.1	13.6	3.8 × 10^–6^
	**Δ**SPO2626	Succinate	homology	92.6	11.0	3.6 × 10^–11^	64.9	5.3	1.9 × 10^–9^
	**Δ**SPO2628	Fumarate	homology	96.7	3.3	2.7 × 10^–14^	95.0	7.9	2.1 × 10^–9^
	**Δ**SPO2628	Malate	homology	11.2	25.3	N.S.	47.7	13.6	1.7 × 10^–5^
	**Δ**SPO2628	Succinate	homology	75.2	11.0	6.9 × 10^–10^	50.8	5.3	9.6 × 10^–9^
	**Δ**SPO2630	Fumarate	homology	96.9	3.3	2.7 × 10^–14^	94.9	7.9	2.1 × 10^–9^
	**Δ**SPO2630	Malate	homology	12.7	25.3	N.S.	62.7	13.6	2.1 × 10^–6^
	**Δ**SPO2630	Succinate	homology	81.8	11.0	2.3 × 10^–10^	58.0	5.3	4.4 × 10^–9^
*cntTUVW*	**Δ**SPO2995	Carnitine	novel	48.1	25.8	1.4 × 10^–3^	69.2	36.7	2.8 × 10^–3^
	**Δ**SPO2996	Carnitine	novel	61.1	25.8	2.5 × 10^–4^	85.7	36.7	9.1 × 10^–4^
*cuyTUVW*	**Δ**SPO3040	Cysteate	novel	50.3	15.3	1.9 × 10^–5^	38.9	30.7	1.9 × 10^–2^
	**Δ**SPO3041	Cysteate	novel	81.3	15.3	3.4 × 10^–9^	40.0	30.7	1.7 × 10^–2^
*dmdT*	**Δ**SPO3186	DMSP	homology	48.9	13.4	1.1 × 10^–4^	33.6	2.4	1.6 × 10^–6^
*potFGHI*	**Δ**SPO3469	Cadaverine	expression	62.3	7.4	8.3 × 10^–7^	61.2	23.5	1.9 × 10^–3^
	**Δ**SPO3469	Putrescine	expression	68.8	10.3	3.2 × 10^–6^	65.1	12.8	1.5 × 10^–4^
	**Δ**SPO3469	Spermidine	expression	63.6	16.8	8.9 × 10^–5^	75.2	5.8	2.4 × 10^–6^

Column headings: Prediction, previous annotation status of the transporter as follows: novel = annotation was neither known nor hypothesized; homology = annotation was hypothesized based on sequence similarity; expression = annotation was hypothesized based on gene expression data; control = annotation was known based on a previous *R. pomeroyi* knockout mutant. ΔOD, percent decrease in optical density of the isolated mutant relative to pooled-RB-TnSeq library with associated 95% confidence interval and *p* value (*n* = 4, ANOVA with TukeyHSD). ΔDrawdown, percent decrease in drawdown by the isolated mutant relative to the pooled-RB-TnSeq library with associated 95% confidence interval and *p* value (*n* = 3, ANOVA with TukeyHSD). GlcNAc, *N*-acetylglucosamine, N.S., not significant (*p* ≥ 0.05).

### Metabolite drawdown screen

For each mutant-substrate pair identified from the growth screens, 3 replicate 220 µl cultures were prepared in 96 well plates by inoculating 3 μl of washed (3x) overnight mutant cultures into minimal medium containing the candidate substrate at 8 mM carbon. Cultures were grown shaking at 25 ^o^C for 24 h or 36 h, depending on the growth rate supported by the carbon source. At termination, 200 µl of medium were collected and centrifuged at 3700 rpm for 10 min, and the supernatant was stored at −80 ^o^C. Metabolite analysis was performed using a Bruker Avance lll 600 MHz spectrometer (Bruker, Billerica, MA, USA) equipped with a 5-mm TCI cryoprobe. Samples were prepared with addition of a deuterated phosphate buffer (30 mmol L^−1^, pH 7.4) and the internal standard 2,2-dimethyl-2-silapentane-5-sulfonate-d_6_ (DSS, 1 mmol L^−1^) (10:1 vol:vol) and transferred to 3 mm NMR tubes (Bruker). Data were acquired by a one dimensional ^1^H experiment with water suppression (noesypr1d, Bruker) at 298 K using TopSpin 3.6.4 (Bruker). For glycerol, a ^1^H *J*-resolved experiment (jresgpprqf) was used to avoid overlapping background peaks. Spectra were processed using NMRPipe on NMRbox [47, 48], and the processed data were analyzed using Metabolomics Toolbox (https://github.com/artedison/Edison_Lab_Shared_Metabolomics_UGA) and MATLAB R2022a (MathWorks). For quantification of metabolites, spectra were normalized to the DSS standard and peak area for representative peaks was calculated. TopSpin experiment settings, NMRpipe spectra processing parameters, and MATLAB data analysis scripts are available in Metabolomics Workbench (see Data Availability).

### Pooled-RB-TnSeq experiment

Minimal medium was prepared for 10 substrates at 8 mM carbon in a 96 well plate, 180 μl per well (*n* = 4). Each well was inoculated with 20 μl of washed (3x) overnight culture of the *R. pomeroyi* pooled-RB-TnSeq library. After growth with shaking at 25 ^o^C for 72 h, cultures were serially transferred into fresh media three times. After the third transfer, the full culture volume of each well (200 μl) was transferred to 800 μl of minimal medium with substrate for a final 72 h grow out at 25 ^o^C. The libraries averaged 15.5 generations under selection (ranging from 13.5 for xylose to 18.5 for *N*-acetylglucosamine). These 1 ml cultures were then transferred to 1.5 ml tubes, pelleted by centrifugation at 8000 × *g* for 3 min, and stored at −80 ^o^C until further processing. Genomic DNA was extracted from the cell pellets using the DNEasy blood and tissue kit (Qiagen, Hilden, Germany). PCR amplification of RB-TnSeq barcodes was performed using primers modified from Wetmore et al. [26] with PhusionHF master mix (Fisher, Pittsburgh, PA). An aliquot of 8 ng of product from each sample was pooled, purified using HiPrep beads (MagBio, Gaithersburg, MD, USA), and sequenced on a NextSeq SE150 Mid Output flow cell (SE150) at the Georgia Genomics and Bioinformatics Core Facility (Athens, Georgia, USA). The initial processing and demultiplexing of sequence data was performed using Perl scripts (MapTnSeq.pl, DesignRandomPool.pl, and MultiCodes.pl) provided in Wetmore et al. [26]. Following quality control, an average of 35,090 unique barcodes mapped to insertions that fell within the interior 10 to 90% of *R. pomeroyi* coding sequences. In total, 55 million reads were mapped to insertions in 3048 genes (out of 4284 protein-encoding genes in the *R. pomeroyi* genome) with a median of 404,513 mapped reads per sample. Further processing of demultiplexed reads was performed in R v3.6.1. Reads mapping to different insertion sites within the same coding sequence were pooled for subsequent analyses. Two sample T tests with multiple comparison adjustment (FDR) were used to identify enrichment or depletion of normalized reads for each transporter on a given substrate relative to all other substrates tested, a modification of the reference medium method for RB-TnSeq analysis described in Borchert et al. [49]. To display enrichment or depletion of multiple transporters and substrates on the same scale, we calculated the fold change of each transporter’s normalized abundance for a given substrate relative to the normalized abundance of that transporter on all other substrates.

### Transporter expression during a Monterey Bay bloom

Processed *R. pomeroyi* transcriptome data (transcripts per million and Z-scores), metadata, and complete experimental methods are available elsewhere [9]. Briefly, on 14 days over 5 weeks, *R. pomeroyi* cells were added to 350 ml of unfiltered Monterey Bay surface water (*n* = 3). *R. pomeroyi* was inoculated at cell numbers equivalent to that of the natural heterotrophic bacteria. Subsequent sequencing analysis indicated that *R. pomeroyi* transcripts averaged 38% of the bacterial reads in the metatranscriptome datasets [50]. Cells were collected by filtration after 90 min and processed for RNAseq analysis.

### Homologs in the Roseobacter group

Roseobacter strains with complete genomes were selected based on Simon et al. [51]. Phylogenic analysis of the 14 selected strains was carried out with a set of 117 single copy genes using GToTree v1.6.37 [52]. *R. pomeroyi* transporter genes with homologs in the other strains were identified by BLASTp using Diamond v2.0.14.152 [53], threshold: E ≤ 10^−5^ and identity ≥ 60% across the full sequence, and checked with a reciprocal best hits analysis. Data analysis and figure generation was performed with R v3.6.1. Manual checks of gene neighborhoods were performed when BLASTp results showed that multicomponent transporters were missing one or more component genes.

## Results and discussion

**Fig. 1:**
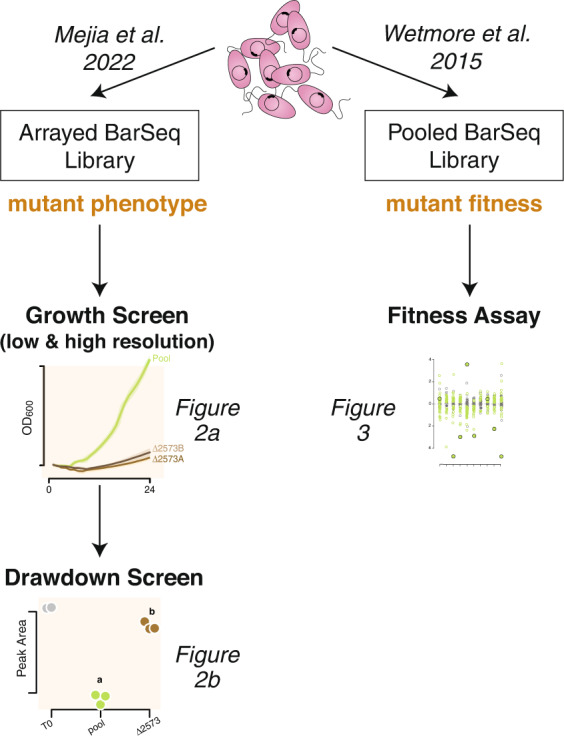
Experimental flow chart for the *R. pomeroyi* RB-TnSeq library. Right path: Pooled mutant populations (pooled-RB-TnSeq library) are used for gene fitness assays. Left path: Individual transporter mutants from the library (arrayed-RB-TnSeq library) are used to screen for growth and metabolite drawdown.

From a pooled-RB-TnSeq transposon mutant library of *R. pomeroyi* prepared according to Wetmore et al. [26], colonies were individually arrayed into 384 well plates (Fig. 1). The gene disrupted in each arrayed mutant was determined by sequencing the transposon barcode in conjunction with indexed primers that indicated plate column and row [38], creating a library that covers 3048 of 4284 protein-encoded genes in the *R. pomeroyi* genome (71%). From the genome annotation [54, 55], the first 156 mutants that were predicted to encode for 104 organic compound influx transporter proteins (Table S1) were re-arrayed into multi-well plates to facilitate functional screens using 70 compounds (Table S2) known to be produced by marine phytoplankton [56].

### Growth screens identify 13 transporters of 18 substrates

**Fig. 2:**
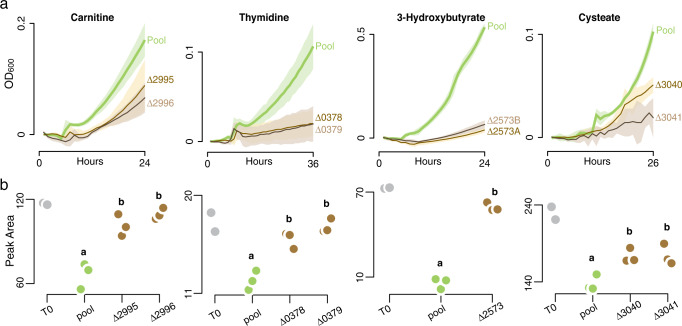
Growth and metabolite drawdown results leading to four novel transporter annotations. **a** Growth of transporter mutants compared to growth of the pooled-RB-TnSeq library (an analog for wild-type growth but carrying transposon and resistance gene insertions) on selected marine plankton metabolites. Shaded regions indicate 95% confidence intervals (*n* = 4). Numbers refer to *Ruegeria pomeroy*i DSS-3 locus tags. **b** Substrate concentrations (^1^H-NMR peak area) after growth of mutants (brown symbols, *n* = 3) or the pooled-RB-TnSeq library (green symbols, *n* = 3), and at inoculation (gray symbols, *n* = 2). Letters that differ indicate that peak areas for the mutants were significantly higher than for the pooled-RB-TnSeq library (ANOVA, *n* = 3, *p* ≤ 0.05), with a TukeyHSD test carried out when multiple mutants for the same substrate were tested (*p* ≤ 0.05). For full results, see Table 1.

Initial screens of the 156 mutants identified candidate substrates of transporter genes based on OD_600_ deficits after 24–72 h (*n* = 2). These mutants were transferred to a second round of screening in which each candidate substrate/mutant pair was monitored for growth with hourly OD readings and higher replication (*n* = 4). A positive control treatment consisting of the pooled-RB-TnSeq library approximated wild-type growth (Fig. 2A, S1). We used mutants of three previously confirmed transporters as positive controls for the screening protocols; these were the *betT* (SPO1087) for choline uptake [42], *hpsKLM* (SPO0591-0593) for DHPS uptake [41, 43], and *uehABC* (SPO1145-1147) for ectoine uptake [44] (Table 1, Fig. S1). One mutant (SPO2952) was excluded from consideration because it exhibited growth defects on multiple structurally dissimilar substrates (alanine, arginine, glycolate, malate, spermidine) in initial growth screens and pooled-RB-TnSeq screens, suggesting a general fitness defect not necessarily related to transport function.

The growth-based screening resulted in substrate predictions for 13 *R. pomeroyi* transporters (Fig. 2A, S1, Table 1). Four of these were consistent with target metabolites hypothesized based on previous gene expression data: *xylFGH* (SPO0861-0863) (glucose/xylose) [57], *iseKLM* (SPO2356-2358) (isethionate) [58], *potFGHI* (SPO3466-3469) (polyamines: cadaverine, spermidine, and putrescine) [59], and *tauABC* (SPO0674-0676) (taurine) [60]. Four were consistent with target metabolites hypothesized based on *in silico* analysis by the GapMind tool for carbon sources, which combines pathway annotations and gap-filling using mutant fitness data:[19] *tctABC* (SPO0184-00186) (citrate), *dctMPQ* (SPO2626-2628) (C4 organic acids succinate, fumarate, and malate), *nagTUVW* (SPO1835, SPO1837-1839) (*N*-acetylglucosamine), and *glpVSTPQ* (SPO0608-0612) (glycerol). One was consistent with a target metabolite based on homology to an experimentally verified transporter in the closely related species *Roseovarius nubinhibens*:[61] *dmdT* (SPO3186) [dimethylsulfoniopropionate (DMSP)] (Table 1). Four were novel annotations with no previous substrate predictions: *cntTUVW* (SPO2995-2998) (carnitine), *cuyTUVW* (SPO3040-3043) (cysteate), *hbtABC* (SPO2571-2573) (3-hydroxybutyrate), and *nupABC* (SPO0376-0379) (thymidine) (Table 1). Previous studies had identified all hypothesized substrates as endometabolites in cultured phytoplankton or natural plankton communities or as exometabolites in phytoplankton cultures or seawater [56, 62–69].

### Metabolite drawdown confirms gene knockout results

Substrate identifications emerging from the growth screens were further tested in metabolite drawdown experiments. Similar to the design of the growth screens, isolated mutants were inoculated into minimal medium with a single substrate as the sole carbon source (*n* = 3), alongside positive control treatments inoculated with the pooled-RB-TnSeq library as an analog for wild type. Spent media samples were collected at 24 h or, for substrates that supported slower growth, at 36-48 h. Substrate concentration was measured by ^1^H-NMR and a mutant drawdown defect was defined as significantly higher substrate concentration in the mutant cultures compared to the pooled-RB-TnSeq library (ANOVA and TukeyHSD, *p* ≤ 0.05, Table 1). All transporter annotations that had emerged from the growth screens were subsequently upheld in these drawdown screens (Fig. 2B, S2), consistent with gene disruption reducing or eliminating substrate uptake (Fig. 2A, S1).

Some transporter mutants, such as *betT*, were completely unable to grow on or draw down the substrate (Figs. S1, S2). This is the expected pattern if the disrupted transporter is the only system for uptake by *R. pomeroyi*. Alternatively, some mutants, such as *dmdT*, were capable of partial growth and drawdown, but significantly less than the mutant pool (Fig. 2, S1, S2). This pattern suggests that more than one transporter in the *R. pomeroyi* genome can take up the compound. For example, *dmdT* belongs to the BCCT-type family whose members frequently have low substrate affinity [70], suggesting to us that a second, high-affinity transporter was available when substrates became depleted. Indeed, a recent paper identified a high affinity DMSP transporter in *R. pomeroyi* (SPO2441-2443; *dmpXWV*) that, like *dmdT*, only partially explained observed DMSP uptake;[71] this second DMSP transporter now brings the number of experimentally verified transporters to 18. In a mixed result, complete loss of growth and drawdown for fumarate yet partial losses for succinate and malate suggests that *dctMPQ* is the only fumarate transporter system in the *R. pomeroyi* genome while the other C4 organic acids have a second transporter (Fig. 2B, S2, Table 1). We note that while we identified three transporters with multiple substrates (C4 organic acids, glucose/xylose, and multiple polyamines), additional multi-substrate transporters would be missed in our analysis if the other target substrate(s) was not among the 70 screened compounds. Further, the efficacy of growth-based screens for identifying transporter substrates is hampered by poor knowledge of the diversity of metabolites that support heterotrophic growth;[56] by the inability to test substrates that don’t support growth as a sole carbon source; by substrates that can be taken up by more than one transporter; and by the limited availability of transporter mutant collections that can facilitate matching transporters with their cognate substrates.

### Pooled-RB-TnSeq studies are consistent with mutant screens

Another approach to identify substrates of bacterial transporters is to place a pooled-RB-TnSeq library under selection on a single carbon source [25]. In this case, transporter mutants that exhibit poor growth are identified as candidate uptake systems. We asked whether the pooled-RB-TnSeq approach would have been sufficient to recognize the *R. pomeroyi* transporters identified here, saving the effort of arraying the RB-TnSeq library while also providing additional information on catabolic and regulatory genes that may support metabolite utilization.

**Fig. 3:**
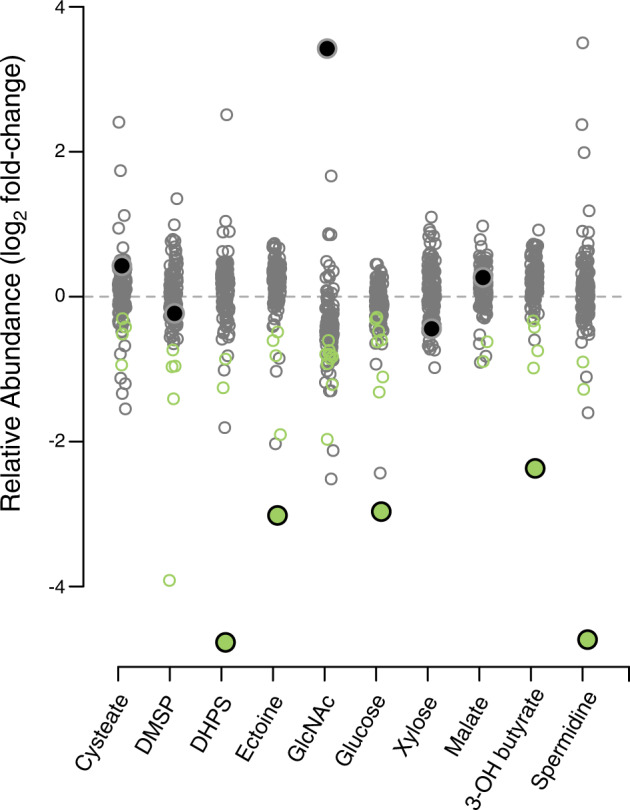
Relative abundance of *Ruegeria pomeroyi* DSS-3 transporter mutants following selection of the pooled-RB-TnSeq library for growth on 10 metabolites. Green symbols indicate significant mutant depletion (*t* test, *n* = 4, Benjamini-Hochberg adjusted *p* ≤ 0.05) and gray symbols indicate non-significant changes. The larger filled symbols indicate the identified transporter for that metabolite as determined from growth and drawdown assays of individual mutants, and is colored green if it was correctly identified, and colored gray if not. Mutant enrichment/depletion for multi-gene transporter systems is plotted as the average of all components.

Mutant abundance was calculated for members of the pooled-RB-TnSeq library following selection for growth on ten substrates used in the growth screens (Fig. 3). Selection occurred over four growth dilution cycles of 72 h each. Amplicon sequencing of the pooled library at the beginning and end of selection [26] was used to calculate relative enrichment/depletion for each mutant in the pool as a proxy for fitness. For five substrates, the pooled RB-TnSeq results agreed with results from the arrayed mutant screens, identifying the same transporter systems for DHPS, ectoine, glucose, 3-hydroxybutyrate, and spermidine (*n* = 4; T test, *p* ≤ 0.05) (Fig. 3). For five other substrates, the known transporter mutant was either not significantly depleted from the mutant pool or significantly enriched, and thus transporters were not correctly identified for DMSP, malate, xylose, cysteate, and *N*-acetylglucosamine. Hypotheses for why these were not identified include the presence of a second transporter in the genome (DMSP, malate), poorer growth leading to weaker selection (xylose), and high substrate concentrations decreasing the need for the transporter substrate binding component (cysteate). In a counterintuitive finding, the *N*-acetylglucosamine transporter mutants (*nagTUVW*) were the most enriched populations in the pool, indicating a fitness gain for cells unable to take up the only provided substrate. We hypothesize that this was due to cross-feeding of an *N*-acetylglucosamine degradation product released by the other mutants and initially used only by the *N*-acetylglucosamine transporter mutant. While these results demonstrate that pooled-RB-TnSeq mutant libraries are excellent tools for low-cost, high-throughput hypothesis generation, predicted transporter annotations nonetheless require experimental follow-up [28, 30].

### Transporter expression reveals the metabolite landscape of a coastal phytoplankton bloom

We used an *R. pomeroyi* gene expression dataset from a natural phytoplankton bloom in Fall 2016 in Monterey Bay, CA, USA [50] to assess the ecological relevance of the verified transporters. On 14 dates over 5 weeks during the decline of a bloom dominated by the dinoflagellate *Akashiwo sanguinea*, *R. pomeroyi* cells were introduced into samples from the natural community for 90 min [9]. Metatranscriptomic data from each sample were subsequently mapped to the *R. pomeroyi* genome to identify environmental conditions eliciting transcriptional responses on each sample date. We reanalyzed this dataset in light of the new information on transporter function, with the goal of generating insights into bloom-associated metabolites supporting heterotrophic bacterial growth.

**Fig. 4:**
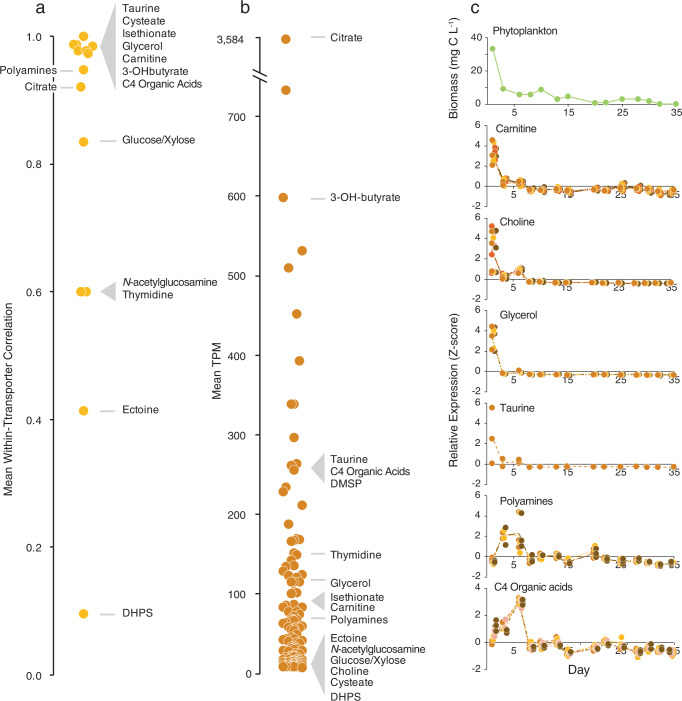
Transporter expression in a Monterey Bay invasion study. **a** Mean correlation coefficients of relative expression levels of genes within multi-component transporter systems in Monterey Bay, in Fall, 2016. **b** Mean relative expression levels for the 126 *R. pomeroyi* transporter systems when introduced into Monterey Bay seawater, averaged across 14 dates. **c** Relative expression of selected *R. pomeroy*i DSS-3 transporters in Monterey Bay seawater on each of 14 dates over 35 days, normalized as Z-scores. Expression of all verified transporters is shown in Fig. S3. Transporters have 1–5 component genes (each colored in different shades of brown) and each component gene has three replicates plotted individually. Lines connect the component mean expression through time. Total phytoplankton biomass (μg C L^–1^) during the 5-week sampling period is also shown.

To first evaluate the internal consistency of the expression data, pairwise correlation coefficients were calculated for the individual components of the 14 multi-component transporters across the sample dates. Nine systems had within-transporter correlation coefficients above 0.84 (Pearson correlation, p ≤ 0.05), confirming coherence in the expression patterns for genes in the same transporter system (Fig. 4a). The remaining four had within-transporter correlation coefficients ranging from 0.10 to 0.60; three of these, however, had particularly low expression in Monterey Bay (Fig. 4b) that may have affected analytical accuracy.

Expression patterns of the carnitine, choline, taurine, and glycerol transporters were positively related to phytoplankton biomass through the bloom (Pearson correlation, *p* ≤ 0.05) (Fig. 4c, S3), and we hypothesize that these compounds were consistently present in the exometabolite pool. Expression of the C4 organic acid transporter and polyamine transporter had peaks coinciding with the largest drop in phytoplankton biomass (Fig. 4c), and we hypothesize that these compounds leaked from senescing or dead phytoplankton. Transcripts from *R. pomeroyi*’s 126 transporter systems were ranked by their abundance in the transcriptomes [mean transcripts per million (TPM), averaged across components for multi-gene transporters]. Making the assumption that heterotrophic bacterial transporter expression is regulated by substrate detection [admittedly an oversimplification [72]], citrate, 3-hydroxybutyrate, taurine, and DMSP, were among the most important sources of organic carbon to *R. pomeroyi* in this dinoflagellate-dominated bloom (expression ranked in the top 25% of transporters). Conversely, DHPS and cysteate were among the least important (ranked in the bottom 25%) (Fig. 4b, S3), both of which are found in endometabolites of diatoms and coccolithophores but not dinoflagellates [64]. The transporter for 3-hydroxybutyrate was of particular interest for two reasons. First, this newly annotated *hbtABC* is the only confirmed bacterial uptake system for 3-hydroxybutyrate [73]. Second, *hbtABC* was the third-most highly expressed *R. pomeroyi* transporter in Monterey Bay. While 3-hydroxybutyrate is well studied as the monomer of the bacterial storage compound polyhydroxybutanoate [74], it is not recognized as an ecologically-important component of the marine dissolved organic carbon pool. This is also the case for citrate, whose transporter was the most highly expressed of all the *R. pomeroyi* transporters (~5-fold higher than the second highest; Fig. 4b). Previous research showed that this central metabolite of the tricarboxylic acid (TCA) cycle and precursor for amino acid and cofactor biosynthesis was among the more abundant metabolites measured in phytoplankton cells in North Pacific surface seawater [62]. Thus its source in Monterey Bay is likely to be the ongoing phytoplankton bloom.

### Orthologous transporters are present in other Roseobacter group members

**Fig. 5:**
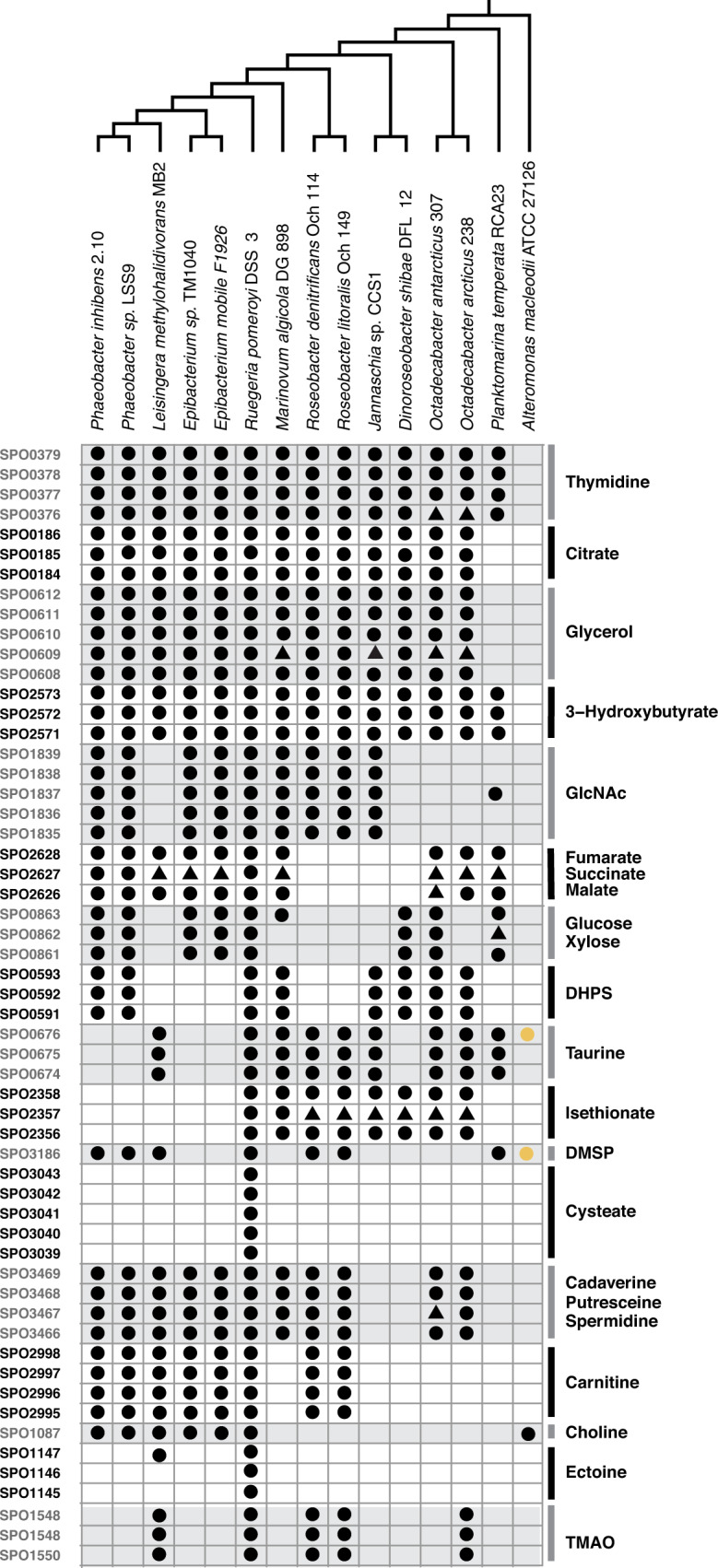
Orthologs of the verified *R. pomeroyi* DSS-3 transporter systems in Roseobacter group members. Each row indicates a single gene and shading groups the genes that make up multi-component transporters. Black circles denote orthologs identified by BLASTp using e ≤ 10^-5^, identity thresholds ≥60%, and reciprocal best hit analysis. Black triangles denote orthologs of multicomponent transporters that did not meet the BLAST thresholds but were co-located in a transporter operon with components that did. Yellow circles denote potential orthologs in the outgroup *A. macleodii* ATCC27126 (identities <40%). Species phylogeny is based on analysis of 117 single copy genes.

*R. pomeroyi* and its relatives in the Roseobacter group are recognized for their high abundance in coastal marine environments [75, 76]. The cultured members of this group typically have large, well-regulated genomes capable of diverse metabolisms [77] and are often associated with phytoplankton blooms [78, 79]. To determine the distribution of the 17 verified transporters in Roseobacter genomes, 13 other strains having closed genomes and representing a broad sampling of the group’s phylogenetic diversity [51] were selected for analysis. Transporters for *N-*acetylglucosamine and carnitine are present only in close relatives of *R. pomeroyi*, consistent with vertical transmission (Fig. 5). Transporters for the organic sulfur compounds DHPS, taurine, and isethionate are common in deeply branching strains but retained in few lineages. The transporters for cysteate and ectoine are unique or nearly so to *R. pomeroyi*, suggestive of specialized niche dimensions. Finally, transporters for thymidine, citrate, glycerol, and 3-hydroxybutyrate are well conserved throughout Roseobacter genomes (Fig. 5), indicating broad importance of these substrates to the ecology of this group. Patchy distribution of transporter orthologs relative to the Roseobacter phylogeny has been reported previously [80].

## Conclusions

Thirteen *R. pomeroyi* transporter annotations were confirmed in a screen of 70 metabolites against 156 transporter mutants representing 104 of the bacterium’s 126 organic carbon influx transporter systems. The verified gene functions provided new insights into in a longitudinal dataset of *R. pomeroyi* transcription through a natural phytoplankton bloom, revealing details of the metabolite landscape and generating hypotheses that citrate, 3-hydroxybutyrate, taurine, and DMSP were highly available metabolites during the dinoflagellate-dominated Monterey Bay bloom. Comparative analysis of the verified transporters across Roseobacter genomes revealed, on the one hand, narrow niche dimensions restricted to subgroups (*R. pomeroyi* and its closest relatives), and on the other, broad ecological characteristics common across the group and reflecting core ecological roles. As is the case for many marine bacterial taxa [81], the streamlined Roseobacter species that are more numerous in ocean microbial communities are poorly represented in culture collections [82]. As such, experimental gene annotation is central for analyzing, or re-analyzing, microbial gene, transcript, and protein data that harbor extensive untapped knowledge. For model organism *R. pomeroyi*, this study brings the percent of organic compound influx transporters with identified substrates to 14% of the 126 gene systems that acquire metabolites from the ocean’s organic carbon pools.

## Supplementary information

Supplemental Figures S1,S2,S3

Supplemental Table S1

Supplemental Table S2

## Data Availability

All growth and RB-TnSeq data are available through BCO-DMO project 884792. All raw NMR data, processing scripts, and processed files for the metabolite drawdown experiment are available in Metabolomics Workbench with Study ID ST002381 (10.21228/M8ST4T).
